# Safety of Triple-Dose Rifampin in Tuberculosis Treatment: A Systematic Review and Meta-Analysis

**DOI:** 10.1093/cid/ciaf004

**Published:** 2025-01-09

**Authors:** Omri A Arbiv, Thomas Holmes, Marie JeongMin Kim, Marie Yan, Kamila Romanowski, Sarah K Brode, William J Burman, Dick Menzies, James C Johnston

**Affiliations:** Faculty of Medicine, University of Toronto, Toronto, Ontario, Canada; Institute of Health Policy, Management, and Evaluation, Dalla Lana School of Public Health, University of Toronto, Toronto, Ontario, Canada; Clinician-Investigator Program, University of British Columbia, Vancouver, British Columbia, Canada; School of Medicine, Royal College of Surgeons in Ireland, Dublin, Ireland; Department of Medicine, University of British Columbia, Vancouver, British Columbia, Canada; Department of Medicine, University of British Columbia, Vancouver, British Columbia, Canada; Provincial TB Services, BC Centre for Disease Control, Vancouver, British Columbia, Canada; Provincial TB Services, BC Centre for Disease Control, Vancouver, British Columbia, Canada; Faculty of Medicine, University of Toronto, Toronto, Ontario, Canada; Division of Respirology, University Health Network, Toronto, Ontario, Canada; Public Health Institute at Denver Health, Denver, Colorado, USA; McGill International TB Centre, McGill University, Montreal, Quebec, Canada; Department of Epidemiology, Biostatistics and Occupational Health, McGill University, Quebec, Canada; Montreal Chest Institute, McGill University Health Centre, Montreal, Quebec, Canada; Department of Medicine, University of British Columbia, Vancouver, British Columbia, Canada; Provincial TB Services, BC Centre for Disease Control, Vancouver, British Columbia, Canada; McGill International TB Centre, McGill University, Montreal, Quebec, Canada

**Keywords:** tuberculosis, rifampin, meta-analysis, systematic review, epidemiology

## Abstract

**Background:**

Recent studies suggest that triple-dose rifampin (TDR; ≥30 mg/kg/d) may be unsafe. We updated a systematic review to investigate the safety and efficacy of TDR.

**Methods:**

We searched Embase, MEDLINE, Cochrane CENTRAL, Cochrane Database for Systematic Reviews, and clinicaltrials.gov for randomized, controlled trials from 1 January 1965 to 10 February 2024 that compared standard-dose rifampin (SDR) with TDR and/or double-dose rifampin (DDR) in human tuberculosis treatment. The primary outcome was pooled incidence rate ratio (IRR) of severe adverse events (SevAEs) between participants who received TDR and those who received SDR. Pooled relative risk (RR) of death was a key secondary outcome. Meta-analysis was performed using the inverse variance method. Heterogeneity was assessed using *I*^2^, and bias was assessed using Cochrane Risk of Bias 2. The protocol was prospectively registered (osf.io/kfn5a).

**Results:**

Of the 11 315 articles identified, 17 met inclusion criteria, enrolling 2313 SDR participants (17 studies), 2238 receiving DDR (12 studies), and 1199 receiving TDR (11 studies). Six studies had a high risk of bias. There was an increase in pooled SevAEs among participants who received TDR compared with those who received SDR (IRR, 1.48; 95% confidence interval [CI], 1.12–1.96; *I*^2^, 23%), driven by hepatic events (IRR, 1.96; 95% CI, 1.21–3.18). Death did not differ between participants who received TDR and SDR (RR, 1.19; 95% CI, .71–1.99). One limitation is that only 2 included studies were blinded.

**Conclusions:**

Regimens that used TDR were associated with an increase in SevAEs, raising concerns regarding safety of TDR regimens.

## Teaser

There is growing interest in high-dose rifampin for tuberculosis treatment. We found that triple-dose rifampin (≥30 mg/kg/d) was associated with an increase in the pooled rate of severe adverse events compared with standard-dose rifampin (10–14 mg/kg/d).

## Graphical Abstract

**Graphical Abstract:**
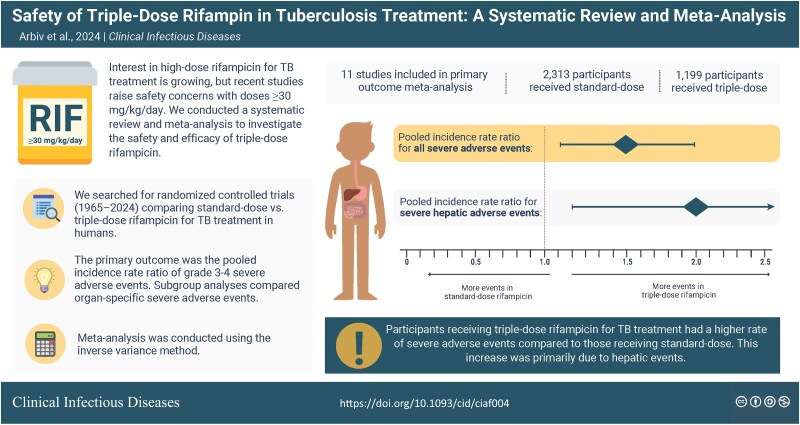

**(See the Review Article by Espinosa-Pereiro et al on pages 129–42; Editorial Commentary by Maranchick and Peloquin on pages 143–4.)**

Since its introduction in the 1960s, rifampin has been a critical component of tuberculosis (TB) drug regimens [1]. When introduced, rifampin dosing was in part curtailed due to financial constraints and concern over adverse drug reactions, including hepatotoxicity, rash, cytopenia, and gastrointestinal distress [2]. Contemporary pharmacokinetic studies have suggested that higher doses of rifampin may have greater antimycobacterial activity [3]. This has spurred numerous clinical trials to investigate the safety and efficacy of high-dose rifampin, particularly with the aim to shorten the duration of pulmonary TB regimens and improve outcomes in TB meningitis [4].

Most early trials that investigated the safety of high-dose rifampin used approximately twice the World Health Organization’s recommended daily dose of 10 mg/kg [5]. A recent meta-analysis of high-dose rifampin data did not identify any significant association between regimens with high-dose rifampin and pooled safety or efficacy end points [4]. Further interest in high-dose rifampin has led to an escalation of rifampin dosing, with minimal harm demonstrated in small dose-finding studies with doses up to 35 mg/kg/d [6]. More recently, however, trials of triple-dose rifampin (TDR) have raised concerns with respect to safety [7, 8]. The pooled outcomes of these studies have yet to be evaluated systematically.

Given the interest and numerous studies that have evaluated TDR [9, 10], we performed a systematic review and meta-analysis to compare the safety and efficacy of regimens that contain TDR with the safety and efficacy of regimens that contain standard-dose rifampin (SDR) for people treated for TB. We also compared the safety and efficacy of double-dose rifampin (DDR) with the safety and efficacy of TDR and SDR. Our primary goal was to determine if there was an increased rate of adverse events (AEs) with regimens that contain TDR compared with SDR.

## METHODS

### Search Strategy and Study Selection

We prospectively registered a protocol at the Open Science Foundation [11]. We searched Embase, MEDLINE, Cochrane Central Registry of Controlled Trials, Cochrane Database for Systematic Reviews, and clinicaltrials.gov, updating our prior review [4]. We limited our search to randomized, controlled trials (RCTs) of rifampin published between 1 January 1965 and 10 February 2024. Search keywords included “rifampicin OR rifampin AND tuberculosis AND randomized controlled trials OR clinical trial”; the complete search strategy is provided in the Supplementary Material (Supplementary Table 1).

We included all studies reported in English that compared oral SDR with DDR and/or TDR in the treatment of TB disease or infection in humans of all ages. We intended to include only studies that measured TDR but subsequently added studies that compared DDR with SDR in order to compare the safety and efficacy across the dosing spectrum. DDR or TDR must have been provided for ≥14 days to be included in the analysis. We excluded studies that did not measure outcomes of interest, were retrospectively collected, or had loss to follow-up of more than 20%. Two reviewers (of O. A., T. H., M. J. K., M. Y., or J. C. J.) independently assessed each title/abstract and full text for inclusion, with adjudication by a third reviewer (J. C. J.) in case of disagreement. Two authors (of O. A., T. H., M. J. K., M. Y., or J. C. J.) extracted relevant study information, including study design, inclusion/exclusion criteria, TB disease site, sample size, AEs, loss to follow-up, withdrawal, culture conversion, and death. Information was obtained from publicly available articles, trial registrations, study protocols, and communications with study authors when warranted. Bias was assessed using the Cochrane Risk of Bias version 2 [12].

### Definitions

TDR was defined as daily oral rifampin ≥30 mg/kg/d, DDR as 15–29 mg/kg/d in adults and 20–29 mg/kg/d in children, and SDR as 10–14 mg/kg/d in adults and 10–19 mg/kg/d in children. The classification of adults and children was study-defined. Severe AEs (SevAEs) were defined as grade 3–4 AEs by Common Terminology Criteria for Adverse Events (CTCAE) or Division of AIDS (DAIDS) Table for Grading the Severity of Adult and Paediatric Adverse Events. If this was not reported, we used AEs that led to cessation of therapy, similar to our prior study [4]. We defined modified Hy's law as an increase of bilirubin ≥2 times the upper limit of normal (ULN) with a coincident increase in alanine or aspartate transaminase ≥3 times ULN [13] unless Hy's law was reported.

### Outcomes

Our primary outcome was the pooled rate of total SevAEs per person-year between participants who received a regimen that contained TDR compared with a regimen that contained SDR. Secondary outcomes included pooled rate of all AE; pooled rates of modified Hy's law; pooled rate of transaminase elevation ≥10 upper limit of normal or >1000 units/L; pooled risk ratios of each of death, 2-month culture conversion, TB relapse, TB recurrence, withdrawal from trial, and loss to follow-up; and pooled hazard ratio of time to culture conversion. TB recurrence was defined as recurrent culture-confirmed TB, whereas TB relapse was defined as TB recurrence with confirmatory genotypic testing. We also compared outcomes in participants who received DDR to SDR or TDR with respect to pooled rate of SevAEs, death, and 2-month culture conversion. We performed a dose-response meta-analysis to compare pooled rates of SevAEs, AEs, and risk of death across rifampin doses.

Subgroup analyses compared organ-specific SevAEs between TDR and SDR groups in each hepatic, nonhepatic gastrointestinal, hematologic, cutaneous, and other organ-specific SevAEs; total SevAEs were compared between TDR and SDR groups with respect to organ affected by TB, TB diagnosis type (microbiologically confirmed diagnosis vs clinical diagnosis), and by adult and pediatric subgroups. We also compared the rate of SevAEs across different lengths of TDR treatment: 14–30, 31–60, 61–90, and >90 days.

We performed 5 ad hoc sensitivity analyses on the primary outcome: comparing study and control arms that did not differ in non-rifampin regimen; removing studies that used high-dose isoniazid with TDR; removing studies with high risk of bias; including only AEs possibly, potentially, or probably related to medications; and excluding studies that exclusively enrolled persons with human immunodeficiency virus (PWH). We also performed a subgroup analysis of hepatic SevAEs, removing hyperbilirubinemia not definitively associated with elevated transaminases, to determine whether hepatic outcomes reflect isolated bilirubin elevation. Supplementary Table 2 summarizes all comparisons.

### Statistical Analyses

Incident rate ratios were created by dividing the number of events by person-years, which were calculated from the median follow-up time and modified intention-to-treat population (mITT). Binary outcomes were analyzed as risk ratio using the mITT population as the denominator. If multiple trial arms met criteria for inclusion in the meta-analysis, arms were pooled [14]. Time-to-culture conversion was assessed using the study-reported adjusted hazard ratio of culture conversion. Random-effects meta-analyses were computed using the inverse variance method with continuity correction. We estimated the proportion of total variability from between-study heterogeneity using *I*^2^ [15]. Linear dose-response meta-analyses were computed using a 2-stage procedure by fitting random-effects models through a restricted maximum-likelihood model [16]. We assessed for publication bias using a funnel plot and Egger's test of the intercept for the primary outcome [17]. Analysis was performed in *R* version 4.3.1 using meta [18] and dosresmeta [19] packages.

## RESULTS

**Figure 1.**
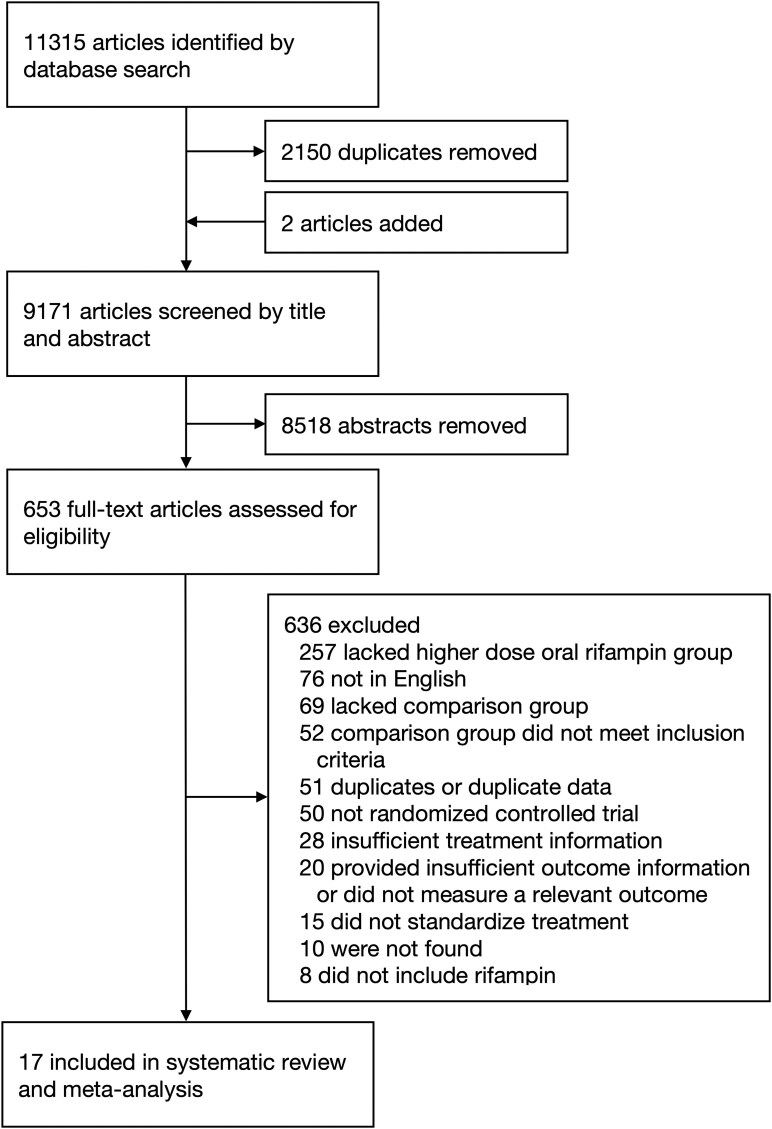
Preferred Reporting Items for Systematic Reviews and Meta-Analyses (PRISMA) diagram.

We identified 11 315 articles (Figure 1), with 653 articles undergoing full-text review and 17 articles included in the systematic review and meta-analysis [7, 8, 20–34], 11 of which included TDR [7, 8, 20–24, 26, 27, 30, 33]. These comprised 15 studies of adults [7, 20–22, 24–34], 1 study in children [23], and 1 study of children and adults [8]. Mean or median age ranged from 25 to 41 years [8, 22, 26–28, 30–34]. Enrollment was limited to those aged ≤65 years in 8 trials [7, 23, 26, 30–34]. Three studies enrolled only PWH [20, 22, 25], while 7 additional studies enrolled at least 1 PWH [24, 26–28, 31, 33, 34]. Several studies excluded participants with chronic kidney disease (CKD) [7, 20–24, 26–28, 30, 31, 34], diabetes or uncontrolled diabetes [7, 21, 26, 32, 34], alcohol use disorder [21, 26], and pregnancy [7, 8, 21, 22, 24–33]. Few studies reported participants with comorbidities, including participants with diabetes [29, 30, 33, 34], alcohol use disorder [30, 33], or CKD [33]. No study reported participant substance use disorder or pregnancy.

Study-level data are available in Table 1. Cumulatively, 1231 SevAEs were recorded across 4462 person-years, including 173 in 970 person-years who received TDR in 11 studies, 539 in 1666 person-years who received DDR in 12 studies, and 519 in 1826 person-years who received SDR in 17 studies. Eleven studies enrolled participants with pulmonary TB [7, 8, 21, 22, 25, 26, 29–32, 34], of which 6 provided TDR [7, 8, 21, 22, 26, 30]; 5 studies of persons with TB meningitis [20, 23, 24, 27, 28], of which 4 provided TDR [20, 23, 24, 27]; and 1 study of TB infection that provided TDR [33]. SevAEs were assessed using DAIDS in 9 studies [7, 20–25, 30, 32] and CTCAE in 6 studies [26–29, 31, 33]; 1 study reported AEs that led to drug cessation [34], and 1 study did not specify grading [8].

**Table 1.** Summary of Studies

Study, Year	Country	Age, y	Tuberculosis Site	Rifampin Dosing	Regimen^a^	N	Severe Adverse Event	Death	Person-Year
Heemskerk et al, 2016 [28]	Vietnam	18-	Meningitis	SDR	R 10 mg/kg, HZE for 3 m; RH for 6 m	409	357	114	302.5
				DDR	R 15 mg/kg, HZE for 2 m; RHZE for 1 m, RH for 6 m	408	404	113	301.8
Jindani et al, 2016 [21]	Bolivia, Nepal, Uganda	18–65	Pulmonary	SDR	R 10 mg/kg, HZE for 8 wk; RH for 18 wk	100	1	0	30.7
				DDR	R 15 mg/kg, HZE for 8 wk; R 15 mg/kg, H for 8 wk; RH for 10 wk	100	3	0	30.7
				DDR	R 20 mg/kg, HZE 8 wk; R 15 mg/kg, H for 8 wk; RH for 10 wk	100	4	1	30.7
Aarnoutse et al, 2017 [31]	Tanzania	18–65	Pulmonary	SDR	R 10 mg/kg, HZE for 2 m; RH for 4 m	50	6	1	11.5
				DDR	R 15 mg/kg, HZE for 2 m; RH for 4 m	50	5	1	11.5
				DDR	R 20 mg/kg, HZE for 2 m; RH for 4 m	50	9	1	11.5
Boeree et al, 2017 [26]	South Africa, Tanzania	23–41	Pulmonary	SDR	R 10 mg/kg, HZE for 8 wk; RH for 18 wk	123	15	0	61.3
				SDR	R 10 mg/kg, HZ, SQ109 300 mg for 12 wk; RH for 14 wk	58	8	0	28.9
				DDR	R 20 mg/kg, HZ, SQ109 300 mg for 12 wk; RH for 14 wk	56	10	0	27.9
				DDR	R 20 mg/kg, HZ, M 400 mg for 12 wk; RH for 14 wk	63	10	0	31.4
				TDR	R 35 mg/kg, HZE for 12 wk; RH for 14 wk	63	10	1	31.4
Dian et al, 2018 [27]	Indonesia	15-	Meningitis	SDR	R 10 mg/kg, HZE 1 m; “standard therapy” for 6 m	20	3	7	9.9
				DDR	R 20 mg/kg, HZE 1 m; “standard therapy” for 6 m	20	8	9	9.9
				TDR	R 30 mg/kg, HZE 1 m; “standard therapy” for 6 m	20	4	3	9.9
Velásquez et al, 2018 [34]	Peru	18–60	Pulmonary	SDR	R 10 mg/kg, HZE for 8 wk; RH for 18 wk	56	2	0	56
				DDR	R 15 mg/kg, HZE for 8 wk; RH for 18 wk	58	1	0	58
				DDR	R 20 mg/kg, HZE for 8 wk; RH for 18 wk	60	6	0	60
Atwine et al, 2020 [25]	Uganda	18-	Pulmonary	SDR	R 10 mg/kg, HZE, EFV 600 mg for 8 wk; RH for 16 wk	33	23	1	17.7
				DDR	R 20 mg/kg, HZE, EFV 600 mg for 8 wk; RH for 16 wk	31	14	1	16.6
				DDR	R 20 mg/kg, HZE, EFV 800 mg for 8 wk; RH for 16 wk	33	16	1	17.7
Maug et al, 2020 [29]	Bangladesh	15-	Pulmonary	SDR	R 10 mg/kg, HZE for 6 m	346	7	11	346
				DDR	R 20 mg/kg, HZE for 6 m	348	3	5	348
Cresswell et al, 2021 [24]	Uganda	18-	Meningitis	SDR	R 10 mg/kg, HZE for 8 wk; RH for 18 wk	21	18	7	9.7
				TDR	R 35 mg/kg, HZE for 8 wk RH for 18 wk	20	11	10	9.2
Paradkar et al, 2022 [23]	India, Malawi	0.5–12	Meningitis	SDR	R 15 mg/kg, HZE for 8 wk; RH for 10 m	14	7	0	16.6
				TDR	R 30 mg/kg, HZE for 8 wk; RH for 10 m	12	16	1	14.3
				TDR	R 30 mg/kg, HZ, LFX 15 mg/kg for 8 wk; RH for 10 m	11	11	0	13.1
Davis et al, 2023 [20]	South Africa	18-	Meningitis	SDR	R 10 mg/kg, HZE for 8 wk; RH for 7 m	20	9	3	9.9
				TDR	R 35 mg/kg, HZE, LZD (1200 mg for 4 wk, 600 mg for 4 wk) for 8 wk; RH for 7 m^b^	14	11	1	6.9
				TDR	R 35 mg/kg, HZE, ASA 1000 mg, LZD (1200 mg for 4 wk, 600 mg for 4 wk) for 8 wk; RH for 7 m^b^	16	13	5	7.9
Jindani et al, 2023 [21]	Botswana, Uganda, Guinea, Nepal, Pakistan, Peru	18-	Pulmonary	SDR	R 10 mg/kg, HZE for 2 m; RH for 4 m	191	9	5	279.8
				DDR	R 20 mg/kg, HZE for 2 m; R 20 mg/kg, H for 2 m	192	10	8	276.6
				TDR	R 30 mg/kg, HZE for 2 m; R 30 mg/kg, H for 2 m	195	10	3	268
Paton et al, 2023 [7]	Indonesia, Philippines, Thailand, Uganda, India	18–65	Pulmonary	SDR	R 10 mg/kg, HZE for 8 wk; RH for 16 wk	181	31	3	333.2
				DDR	R 20 mg/kg, LZD 600 mg, HZE for 8 wk^c^	96	19	NR	176.7
				TDR	R 35 mg/kg, LZD 600 mg, HZE for 8 wk^c^	88	22	NR	162
				TDR	R 35 mg/kg, CFZ 200 mg, HZE for 8 wk^c^	78	14	0	143.6
Sekaggya-Wiltshire et al, 2023 [22]	Uganda	18-	Pulmonary	SDR	R 10 mg/kg, HZE for 8 wk, DTG 50 mg BID; RH for 16 wk,	33	2	0	15.2
				SDR	R 10 mg/kg, HZE for 8 wk, EFV 600 mg daily; RH for 16 wk	34	4	0	15.6
				TDR	R 35 mg/kg, HZE for 8 wk, DTG 50 mg BID; RH for 16 wk	31	7	2	14.3
				TDR	R 35 mg/kg, HZE for 8 wk, EFV 600 mg daily; RH for 16 wk	30	5	2	13.8
Souleymane et al, 2023 [8]	Niger	All	Pulmonary	SDR	R 10 mg/kg, HZE for 2 m; RH for 4 m	65	2	1	32.1
				TDR	R 30 mg/kg, H 15 mg/kg, ZE for 2 m; RH for 4 m	62	11	4	30.6
Kannabiran et al, 2024 [30]	India	18–60	Pulmonary	SDR	R 10 mg/kg, HZE for 2 m; RHZE for 4 m	105	10	3	105
				DDR	R 25 mg/kg, HZE for 2 m; RHZE for 4 m	112	11	1	112
				TDR	R 35 mg/kg, HZE for 2 m; RHZE for 4 m	106	13	2	106
Ruslami et al, 2024 [33]	Canada, Indonesia, Vietnam	10–65	Infection	SDR	R 10 mg/kg for 4 m	454	5	0	145
				DDR	R 20 mg/kg for 2 m	461	6	2	145
				TDR	R 30 mg/kg for 2 m	453	15	0	139

Abbreviations: ASA, aspirin; BID, twice daily; CFZ, clofazimine; DDR, double-dose rifampin; DTG, dolutegravir; E, ethambutol; EFV, efavirenz; H, isoniazid; HZ, isoniazid, pyrazinamide; HZE, isoniazid, pyrazinamide, ethambutol; LFX, levofloxacin; LZD, linezolid; M, moxifloxacin; NR, not reported; R, rifampin; RH, rifampin, isoniazid; RHZE, rifampin, isoniazid, pyrazinamide, ethambutol; SDR, standard-dose rifampin; SevAE; severe adverse event; TDR, triple-dose rifampin; Z, pyrazinamide; ZE, pyrazinamide, ethambutol.

^a^Dosing per accepted international standards where doses are not indicated. All doses listed as oral daily equivalent unless otherwise stated.

^b^Fourteen participants were randomized in a nested trial to receive 3 days of rifampin 20 mg/kg IV.

^c^Treatment extended for an additional 4 weeks if persistent clinical disease at 8 weeks.

One study reported on Hy's law, with 2 participants of 225 receiving TDR, 1 participant of 223 receiving DDR, and no participants of 224 receiving SDR that met Hy's law criteria [21]. Two additional participants who received TDR met Hy's law criteria but tested positive for hepatitis B surface antigen [21]. Two studies reported that participants met laboratory criteria for Hy's law. Dian et al identified 1 participant in 20 who received TDR with transaminase elevation ≥3 ULN and bilirubin elevation ≥10 times ULN, with resolution upon change to SDR [27]. Kannabiran et al noted 2 participants of 105 who received SDR, 5 of 112 who received DDR, and 10 of 106 who received TDR who met laboratory criteria for Hy's law [30].

Risk of bias is summarized in Table 2 and was high in 6 studies, low in 7 studies, and with some concerns in 4 studies. Of studies that assessed the primary outcome, only 2 were blinded and placebo-controlled [26, 27], with 1 trial reporting blinded adjudication of AEs [33]. All other trials that assessed the primary outcome were open-label. There was no evidence of publication bias for the primary outcome by Egger's test of the intercept (*t*(9) = 1.80, *P* = 0.12). Visual inspection of the funnel plot was not suggestive of publication bias (Supplementary Figure 1).

**Table 2.** Risk of Bias by Cochrane Risk of Bias Version 2

	Domain 1	Domain 2	Domain 3	Domain 4	Domain 5	
Study, y	Randomization Process	Deviation From Intended Intervention	Missing Outcome Data	Measurement	Selection of Reported Result	Total Score
Heemskerk et al, 2016 [28]	Low	Low	Low	Low	Low	Low
Jindani et al, 2016 [21]	Low	Low	Low	Low	Low	Low
Aarnoutse et al, 2017 [31]	Low	Low	Low	Low	Low	Low
Boeree et al, 2017 [26]	Low	Low	Low	Low	Low	Low
Dian et al, 2018 [27]	Low	Low	Low	Low	Low	Low
Velásquez et al, 2018 [34]	Low	Low	Low	Low	Low	Low
Atwine et al, 2020 [25]	Low	Low	Low	Low	Low	Low
Maug et al, 2020 [29]	Some concerns	Low	Low	Low	Low	Some concerns
Cresswell et al, 2021 [24]	Low	Low	Low	Some concerns	Low	Some concerns
Paradkar et al, 2022 [23]	Some concerns	Low	Low	Some concerns	Low	High
Davis et al, 2023 [20]	Low	Low	Some concerns	Some concerns	Low	High
Jindani et al, 2023 [21]	Low	Low	Low	Some concerns	Low	Some concerns
Paton et al, 2023 [7]	Low	High	Low	Some concerns	Low	High
Sekaggya-Wiltshire et al, 2023 [22]	Low	Low	Some concerns	Some concerns	Low	High
Souleymane et al, 2023 [8]	Some concerns	Some concerns	Low	High	Low	High
Kannabiran et al, 2024 [30]	Low	Low	Low	Some concerns	High	High
Ruslami et al, 2024 [33]	Low	Low	Low	Some concerns	Low	Some concerns

**Figure 2.**
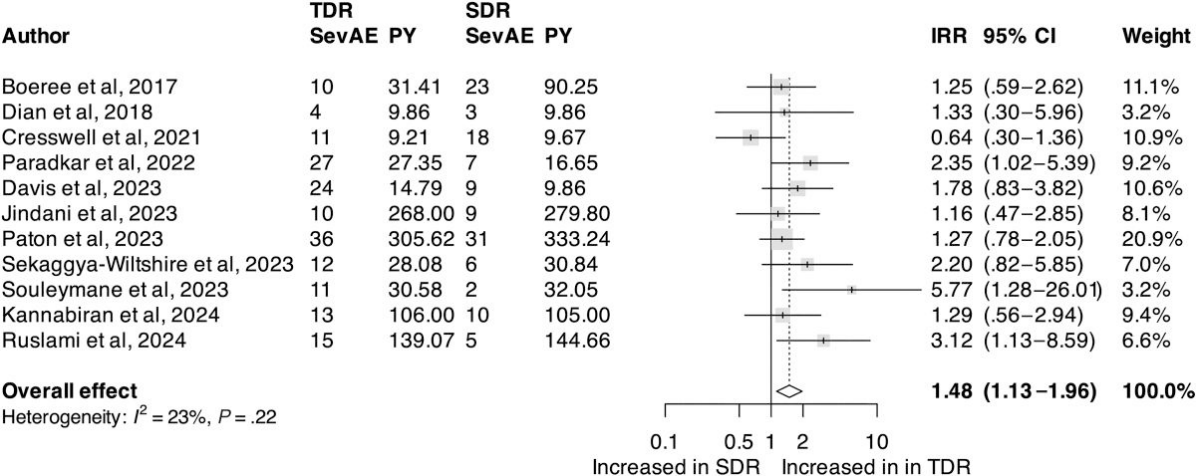
Forest plot of the primary outcome. Comparison of pooled IRR of SevAEs in participants who received regimens that contained TDR compared with SDR. Abbreviations: CI, confidence interval; IRR, incidence rate ratio; PY, person-years; SDR, standard-dose rifampin; SevAE, severe adverse event; TDR, triple-dose rifampin.

**Figure 3.**
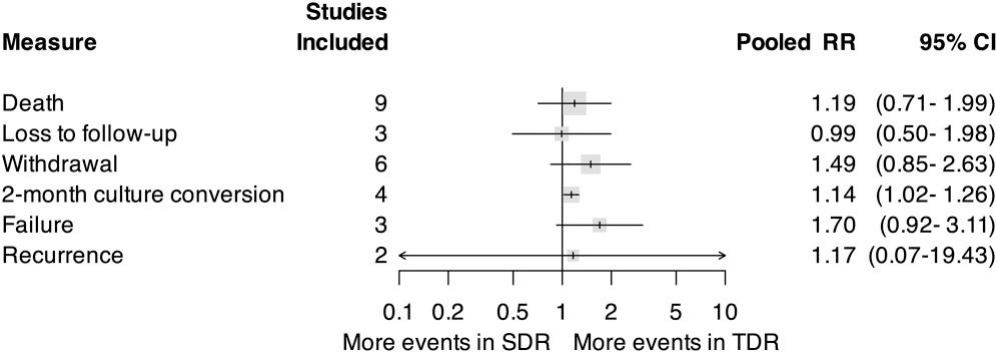
Pooled risk ratio of key secondary outcomes with 95% CIs. Gray box is proportional to the number of studies included. All comparisons consist of pooled RR in participants who received regimens that contained TDR compared with SDR. Abbreviations: CI, confidence interval; RR, risk ratio; SDR, standard-dose rifampin; TDR, triple-dose rifampin.

**Figure 4.**
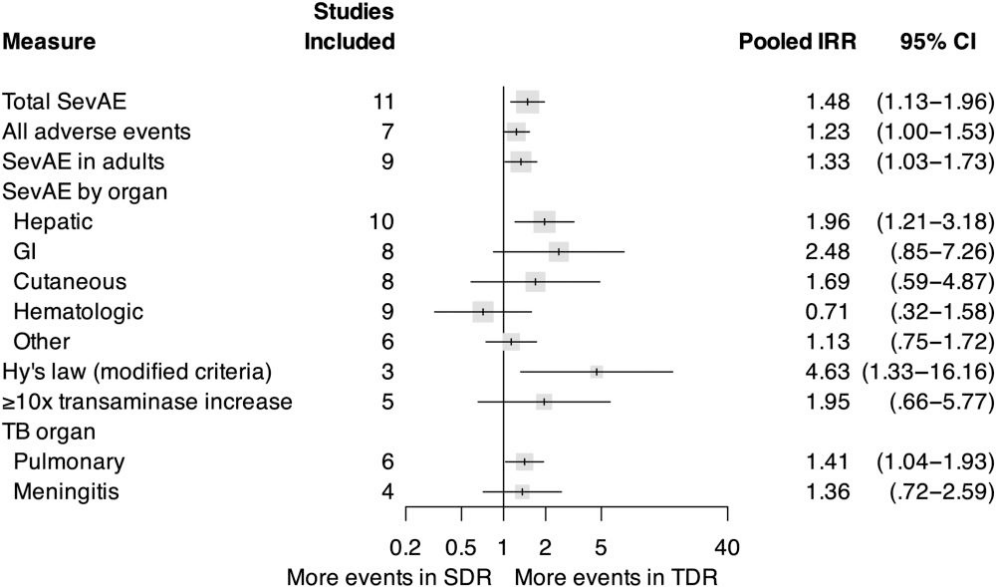
Pooled IRR of key secondary outcomes and subgroup analyses with 95% CIs. Gray box is proportional to the number of studies included. All comparisons consist of IRR in participants who received regimens that contained TDR compared with SDR. Abbreviations: CI, confidence interval; GI, gastrointestinal; IRR, incidence rate ratio; SDR, standard-dose rifampin; SevAE, severe adverse event; TB, tuberculosis; TDR, triple-dose rifampin.

For our primary outcome, we identified an increase in pooled SevAEs among participants who received TDR compared with SDR (incidence rate ratio [IRR], 1.48; 95% confidence interval [CI], 1.12–1.96; *I*^2^, 23%; Figure 2). There was no significant difference in the pooled risk ratio of death (RR, 1.19; 95% CI, .71–1.99; *I*^2^, 0%; Figure 3), loss to follow-up (RR, 0.99; 95% CI, .50–1.99), withdrawal (RR, 1.49; 95% CI, .85–2.63), treatment failure (RR, 1.70; 95% CI, .92–3.11), or disease recurrence (RR, 1.17; 95% CI, .07–19.43). Relapse was reported in 1 trial [21]. There was an increase in pooled 2-month culture conversion among those who received TDR compared with those who received SDR (RR, 1.14; 95% CI, 1.02–1.26). Participants who received TDR were also noted to have a higher pooled rate of participants who met the modified Hy's law compared with those who received SDR (IRR, 4.63; 95% CI, 1.33–16.16). Comparison of pooled AEs between participants who received TDR and those who received SDR did not meet statistical significance (IRR, 1.23; 95% CI, 1.00–1.51; Figure 4), nor did transaminase elevation exceeding ≥10 times upper limit or ≥1000 units/L (IRR, 1.95; 95% CI, .66–5.77).

In subgroup analyses between participants who received TDR and those who received SDR, we identified an increase in pooled hepatic SevAEs (IRR, 1.96; 95% CI, 1.21–3.18; Figure 4). There was no statistically significant difference in pooled nonhepatic gastrointestinal (IRR, 2.48; 95% CI, .85–7.26), cutaneous (IRR, 1.69; 95% CI, .59–4.87), hematologic (IRR, 0.71; 95% CI, .32–1.58), or other SevAEs (IRR, 1.13; 95% CI, .75–1.72). In the subgroup of participants with pulmonary TB, there was a statistically significant increase in the pooled rate of SevAEs (IRR, 1.41; 95% CI, 1.04–1.93), which was not seen in participants with TB meningitis (IRR, 1.36; 95% CI, .72–2.59). A summary of subgroup analyses is presented in Supplementary Table 2. In a dose-response meta-analysis of all studies, we found a linear increase in the pooled rate of SevAEs and all AEs but no change in death with increasing rifampin dose (Supplementary Figures 2 and 3).

We compared pooled rates of SevAEs among participants who received TDR with rates among participants who received DDR and found no significant differences (IRR, 1.13; 95% CI, .82–1.56). There was a nonsignificant trend toward increasing AEs between those who received DDR and those who received SDR (IRR, 1.12; 95% CI, .99–1.26; Supplementary Figure 4A). There were no differences in pooled RR of death in pairwise comparisons between those who received TDR and those who received DDR (RR, 0.48; 95% CI, .22–1.02) or between those who received DDR and those who received SDR (IRR, 1.00; 95% CI, .82–1.22; Supplementary Figure 4B). Two-month culture conversion was greater in participants who received DDR compared with those who received SDR (RR, 1.09; 95% CI, 1.04–1.14; Supplementary Figure 4C) but was not different between participants who received TDR and those who received DDR (RR, 0.99; 95% CI, .94–1.04).

In sensitivity analyses comparing only treatments in which rifampin was varied between the TDR and SDR groups, we found no significant difference in pooled SevAEs (IRR, 1.51; 95% CI, .95–2.39; Supplementary Figure 5). When studies that included triple-dose isoniazid were removed, there was no significant increase in pooled SevAEs between participants who received TDR and those who received SDR (IRR, 1.41; 95% CI, 1.09–1.82). There were no significant differences in our primary outcome after removal of studies with high risk of bias (IRR, 1.26; 95% CI, .73–2.07) or when we included only SevAEs deemed to be at least possibly caused by medications (IRR, 1.81; 95% CI, .70–4.70). When we excluded studies that exclusively enrolled PWH, the increase in pooled SevAEs remained (IRR, 1.54; 95% CI, 1.15–2.05). Last, when we removed isolated hyperbilirubinemia, we found that the increase in pooled hepatic SevAEs remained (IRR, 1.88; 95% CI, 1.16–3.05).

## DISCUSSION

In a systematic review and meta-analysis of RCTs that compared higher-dose rifampin-based regimens with SDR regimens, we found that regimens that contained TDR were associated with a greater rate of SevAEs compared with regimens that contained SDR. This appeared to be driven by hepatic events. Increased efficacy was noted with both TDR and DDR regimens, with increased pooled 2-month culture conversion compared with SDR regimens and decreased time-to-culture conversion with TDR regimens compared with SDR regimens, although there was no change in other efficacy outcomes, including disease recurrence and treatment failure. The increase in pooled SevAEs between TDR and SDR persisted in participants with pulmonary TB but was not statistically significant in persons with TB meningitis, albeit with fewer published studies. Pooled mortality was similar between each rifampin dosing regimen.

There was an increase in participants who met a modified definition of Hy's law when receiving regimens that contained TDR compared with SDR. We identified 13 of 1199 participants who received TDR, 6 of 2238 who received DDR, and 2 of 2313 who received SDR who met this definition. This is particularly concerning since the US Food and Drug Administration notes that finding 2 cases that meet Hy's law is highly predictive of drug-induced liver injury in a larger population [13]. Critically, these toxicities were noted in participants enrolled in RCTs, which often have lower risk of AEs when compared with routine clinical practice [35]. Indeed, we noted that several studies excluded participants with characteristics that placed them at higher risk for AEs, such as CKD, pregnancy, alcohol use disorder, and older age.

We recently evaluated the safety and efficacy of high-dose rifamycins in studies up to 2021 [4] using a similar protocol. In that analysis, we did not identify an increase in pooled rate of SevAEs in participants who received high-dose rifamycin (≥10 mg/kg/d rifampin equivalent) compared with standard-dose rifamycin (10 mg/kg/d rifampin equivalent). Similarly, we did not identify a difference in sputum culture conversion between rifampin dosing groups. We did note an increase in other SevAEs (ie, not hepatic, gastrointestinal, cutaneous, or hematologic) among participants who received high-dose rifamycin-based regimens compared with standard dose-rifamycin. Interestingly, in this updated analysis, the increase in the rate of other SevAEs with high-dose rifamycin did not hold, suggesting that this was a statistically anomalous finding.

There are several strengths in this study. We performed the review using a prespecified protocol with clear primary and secondary outcomes and subgroup analyses. We used multiple measures of safety and efficacy in our analyses. We also included a wide variety of studies with participants from 18 countries on 4 continents with varied socioeconomic backgrounds. Using these multiple primary and secondary outcomes along with sensitivity analyses, we saw consistent effects in safety and efficacy.

There are several important limitations in our study. First, there is significant heterogeneity across studies in terms of setting, participant populations, and reporting. Where possible, we worked to align data by examining study protocols and study materials to supplement our understanding and contacted study authors when necessary. Second, there were several trials with additional antituberculous medications in experimental treatment regimens along with TDR, which were not present in the SDR control arm. In response, we performed a sensitivity analysis to compare studies where only rifampin dosing varied between experimental and control arms. This comparison was not statistically significant, possibly due to reduced statistical power; however, the trend aligned with our primary outcome. Although we sought to include participants of all ages and with varying TB organ involvement, we identified only 1 study of TB infection, 1 study of children, and 1 study of both children and adults. The generalizability of our findings is therefore more limited in those populations. Finally, only 2 studies were blinded, and the majority of included studies were open-label in design, without blinded adjudication of AEs, which may bias evaluation of AEs.

Overall, in a meta-analysis of 17 RCTs, 11 of which included TDR, we determined that regimens that contained TDR were associated with a greater pooled rate of SevAEs. There were signs of increased efficacy in both TDR and DDR regimens compared with SDR regimens, although this must be balanced with risk of hepatotoxicity. Our meta-analysis suggests that more studies are needed to evaluate DDR, as the limited evidence available suggests a possible increase in AEs among participants who receive DDR. There are several ongoing studies evaluating regimens that contain TDR [9, 10] that are concerning in light of these results. Our findings suggest that use of TDR in clinical and potentially experimental settings should be reassessed, given its potential for increased toxicity.

## Supplementary Material

ciaf004_Supplementary_Data

## Supplementary Data

Supplementary materials are available at *Clinical Infectious Diseases* online. Consisting of data provided by the authors to benefit the reader, the posted materials are not copyedited and are the sole responsibility of the authors, so questions or comments should be addressed to the corresponding author.

## Notes

***Author Contributions.*** J. C. J., D. M., and W. B. initiated the project and were responsible for the design of the protocol. O. A. A., T. H., M. K., M. Y., and J. C. J. reviewed the literature, collected the data, and assessed the quality of the studies. O. A. A. analyzed the data. O. A. A. and J. C. J. interpreted the data. O. A. A. wrote the initial draft of the manuscript. M. Y., K. R., S. K. B., W. B., and D. M. critically revised the manuscript and provided important intellectual content. All authors approved the final version submitted for publication.

***Financial support***. This work was supported by the University of British Columbia Clinician-Investigator Program to O. A. A., the Canadian Institute of Health Research to D. M., and the Health Research British Columbia Health Professional Investigator Program to J. C. J.

***Data sharing*.** Extracted data and code are available from the corresponding author on request.
