# Supplementary material for: Safety of Triple-Dose Rifampin in Tuberculosis Treatment: A Systematic Review and Meta-Analysis
Source: Clin Infect Dis. 2025 Jan 9;81(1):119–28. doi: 10.1093/cid/ciaf004 (PMC12314497; doi:10.1093/cid/ciaf004)
Supplement: ciaf004_Supplementary_Data [file ciaf004_supplementary_data.pdf]

Supplementary Appendix

Table of contents:

Extraction sheet 1-2
Table S1: Search strategy 3
Table S2: Summary of assessed outcomes 4-5
Figure S1 6
Figure S2 7
Figure S3 8
Figure S4 9
Figure S5 10

Extraction sheet

- First author
- Year of publication
- Study title
- Journal
- Other studies published using the same cohort
- Study design
- Countries from which data was collected
- Year in which the study was initiated
- Year in which the study was completed
- Funding source
- Industry involvement
- Active TB
- Organ with TB, for active TB
- Method of TB diagnosis
- TB treatment resistance
- Treatment phase (intensive, continuation)
- Were the patients previously treated for TB
- Trial randomization
- Allocation concealment
- Type of blinding, if applicable
- Percent lost to follow-up or withdrawal
- Group comparability at baseline, generally
- Group comparability at baseline, details
- Directly observed therapy (DOT) or self-administered therapy
- Type of DOT (e.g., pharmacist, nurse)
- Follow-up period
- Inclusion criteria
- Exclusion criteria
- Minimum age of inclusion
- Maximum age of inclusion
- Median age of inclusion
- Mean age of inclusion
- Mean BMI
- Median BMI
- Percent with low BMI
- Treatment regimens
    - Type of rifamycin provided
    - Dose of rifamycin
    - Rifamycin dose considered high (see study for details)
    - Rifamycin dose considered to be very high (see study for details)
    - Rifamycin weekly dose
    - Frequency of rifamycin administration per week
    - Treatment provided ≥5 days per week
    - Route of rifamycin administration
    - Isoniazid dose provided
    - Pyrazinamide dose provided
    - Pyridoxine dose provided
    - Ethambutol dose provided
    - Levofloxacin dose provided
    - Streptomycin dose provided
    - Other treatment provided, with doses

- Length of treatment
- Sample size, intention-to-treat
- Sample size, modified intention-to-treat
- Sample size, per-protocol
- Sample size details
- Number of patients with HIV
- Number of patients with diabetes mellitus
- Number of patients with chronic kidney disease
- Number of patients with substance use disorder
- Number of patients with alcohol use disorder
- Trial exclusion of pregnancy
- Number of patients that withdrew from trial
- Number of patients that were lost to follow-up
- Method of counting severe events
- Number of patients that experienced at least one severe adverse event (SevAE)
- Number of total SevAE
- SevAE considered to be possibly, probably, or likely related to medications
- Flu-like SevAE
- Details on flu-like SevAE
- Cutaneous SevAE SevAE
- Details on cutaneous SevAE
- Hepatic SevAE
- Details on hepatic SevAE
- Gastrointestinal SevAE
- Details on gastrointestinal SevAE
- Hematologic SevAE
- Details on hematologic SevAE
- Other SevAE
- Details on other SevAE
- Non-SevAE
- Details non-SevAE
- Negative culture at 1 month
- Negative culture at 2 months
- Negative culture at 3 months
- Negative culture at 4 months
- Negative culture at 5 months
- Negative culture at 6 months
- Unadjusted hazard ratio of culture conversion
- Adjusted hazard ratio of culture conversion
- Number of patients with disease relapse
- Number of patients with recurrence
- Number of patients with treatment failure
- Number of patients who died
- Additional trial details

Table S1: Search strategy

	Searches	MEDLINE	Embase	Cochrane Central Register of Controlled Trials
1	Tuberculosis/	114854	133642	1822
2	Tuberculosis.tl,ab.	216471	215112	6695
3	or/1-2	248839	252315	7023
4	exp Rifamycins/	24304	3886	1986
5	rifabutin*.mp.	2809	7175	267
6	RFB.mp.	862	972	30
7	rifampin*.mp.	24777	12613	267
8	rifampicin*.mp.	20051	108870	1878
9	RMP.mp.	1929	2913	140
10	RIF.mp.	6852	10309	682
11	rifapentine*.mp.	702	2283	233
12	RPT.mp.	903	1394	132
13	rofact*.mp.	0	2	0
14	rimactane*.mp.	18	383	5
15	rifadin*.mp.	63	1114	16
16	rifater*.mp.	37	380	22
17	rifamate*.mp.	5	71	22
18	or/4-17	46369	126921	4
19	random*.mp.	1738084	2306373	4165
20	prospective*.mp.	1090140	1581996	290993
21	Controlled clinical trial/	95551	472383	7
22	Prospective studies/	679597	797950	127267
23	Cross-Over Studies/	56160	69420	47576
24	Randomized controlled trial/	608500	806896	37
25	Single-Blind Method/	33241	69420	27071
26	Double-Blind Method/	177493	190808	169681
27	Clinical Trial/	539504	1079116	10
28	Cohort Studies/	337647	976706	12243
29	(random* or factorial* or crossover* or cross over* or placebo* or (doubl* adj blind*) or (singl* adj blind*) or assign* or allocat* or volunteer*).tw.	2145437	2880409	1405632
30	or/19-29	3674738	5589503	1580046
31	3 and 18 and 30	2614	6788	1248
32	limit 31 to yr="1965 -Current"	2614	6786	1248

Cochrane Database of Systematic Reviews

	Searches	Results
1	Tuberculosis.mp.	357
2	rifabutin*.mp.	13
3	rifampin*.mp.	54
4	rifampicin*.mp.	96
5	rifapentine*.mp.	7
6	rofact*.mp.	1
7	rimactane*.mp.	2
8	rifadin*.mp.	2
9	rifater*.mp.	1
10	rifamate*.mp.	0
11	or/2-10	114
12	1 and 11	50
13	limit 12 to yr="1965 -Current"	41

ClinicalTrial.gov (*Search repeated on multiple occasions)*

Condition or disease
Tuberculosis
Other Terms
Rifamycin OR rifampin OR rifampicin OR rifapentine OR rifabutin
Total: 629

Table S2: Summary of assessed outcomes

**Type of outcome**	**Measure**	**Groups Compared**	**Measure**	**Point Estimate**	**95% CI**	I^2
Primary	Total SevAE	TDR to SDR	IRR	1.48	1.12–1.96	23%
Secondary	AE	TDR to SDR	IRR	1.23	1.00–1.51	56%
	Hy's law	TDR to SDR	IRR	4.63	1.33–16.16	0%
	Transaminase ≥10x ULN or ≥1000	TDR to SDR	IRR	1.95	0.66–5.77	0%
	Death	TDR to SDR	RR	1.19	0.71–1.99	14.9%
	Loss to follow-up	TDR to SDR	RR	0.99	0.50–1.99	0%
	Withdrawal	TDR to SDR	RR	1.49	0.85–2.63	0%
	Treatment failure	TDR to SDR	RR	1.70	0.92–3.11	0%
	Two-month culture conversion	TDR to SDR	RR	1.14	1.02–1.26	49%
	Recurrence	TDR to SDR	RR	1.17	0.07–19.43	78%
	Relapse			Not done (only one study)		
	Time to culture-conversion	TDR to SDR	aHR	1.86	1.47–2.35	0%
	Total SevAE	TDR to DDR	IRR	1.13	0.82–1.56	2.1%
	Total SevAE	DDR to SDR	IRR	1.12	0.99–1.26	0%
	Death	TDR to DDR	RR	0.48	0.22–1.02	13.4%
	Death	DDR to SDR	RR	1.00	0.82–1.22	0%
	Two-month culture conversion	TDR to DDR	RR	0.99	0.94–1.04	22%
	Two-month culture conversion	DDR to SDR	RR	1.09	1.04–1.14	0%
	Comparison of SevAE by treatment duration	TDR to SDR (Figure S5 for details)	IRR	$\chi^2(3) = 0.57$, $p = 0.90$		
	Total SevAE	DRMA	IRR	$\chi^2(1) = 7.05$, $p = 0.008$		
	AE	DRMA	IRR	χ^2 (1) = 4.69, $p = 0.03$		
	Death	DRMA	RR	χ^2 (1) = 0.003, $p = 0.96$		
Subgroup analyses	Hepatic SevAE	TDR to SDR	IRR	1.96	1.21–3.18	5%
	Non-hepatic gastrointestinal SevAE	TDR to SDR	IRR	2.48	0.85–7.26	0%
	Haematologic SevAE	TDR to SDR	IRR	0.71	0.32–1.58	0%
	Cutaneous SevAE	TDR to SDR	IRR	1.69	0.59–4.88	0%
	Other SevAE	TDR to SDR	IRR	1.13	0.75–1.72	0%
	Total SevAE among patients	TDR to SDR	IRR	1.39	1.08–1.78	16%

	with TB disease					
	Total SevAE among patients with pulmonary TB	TDR to SDR	IRR	1.41	1.04–1.93	0%
	Total SevAE among patients with TB meningitis	TDR to SDR	IRR	1.36	0.72–2.59	50%
	Total SevAE among patients with TB infection	TDR to SDR	IRR	Not done (only one study)		
	Total SevAE in studies of adult patients only	TDR to SDR	IRR	1.33	1.03–1.73	1%
	Total SevAE in studies diagnosing TB clinically	TDR to SDR	IRR	1.36	0.72–2.59	50%
	Total SevAE in studies with microbiologic diagnosis of TB	TDR to SDR	IRR	1.41	1.04–1.93	0%
Sensitivity analyses	Total SevAE in studies without high dose isoniazid	TDR to SDR	IRR	1.41	1.09–1.82	7%
	Total SevAE restricting to studies in which only rifampin dose varied	TDR to SDR	IRR	1.51	0.95–2.39	37%
	Total SevAE in studies not classified as high risk of bias	TDR to SDR	IRR	1.23	0.73–2.07	35%
	Total SevAE possibly caused by medications	TDR To SDR	IRR	1.81	0.70–4.70	51%
	Total SevAE in studies that did not exclusively enrol persons living with HIV	TDR to SDR	IRR	1.54	1.15–2.05	5%
	Hepatic SevAE removing hepatic episodes of bilirubin elevation	TDR to SDR	IRR	1.88	1.16–3.05	0%

Abbreviations: AE, adverse events; aHR, adjusted hazards ratio; CI, confidence intervals; DDR, double-dose rifampin; DRMA, dose-response meta-analysis; HIV, human immunodeficiency virus; IRR, incident rate ratio; RR, relative risk; SDR, standard-dose rifampin; SevAE, severe adverse events; TB, tuberculosis; TDR, triple-dose rifampin; ULN, upper limit of normal.

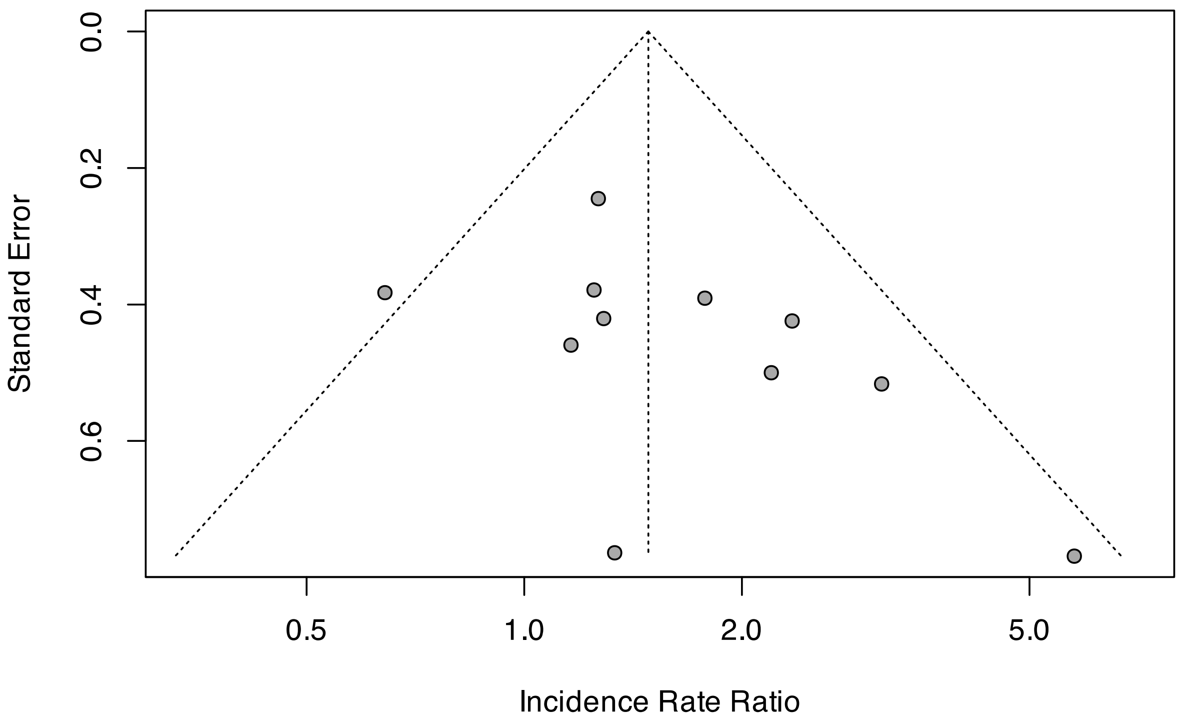

Supplementary Figure 1: Funnel plot for the primary outcome, total severe adverse events, between participants receiving treatment containing triple-dose rifampin and standard-dose rifampin.

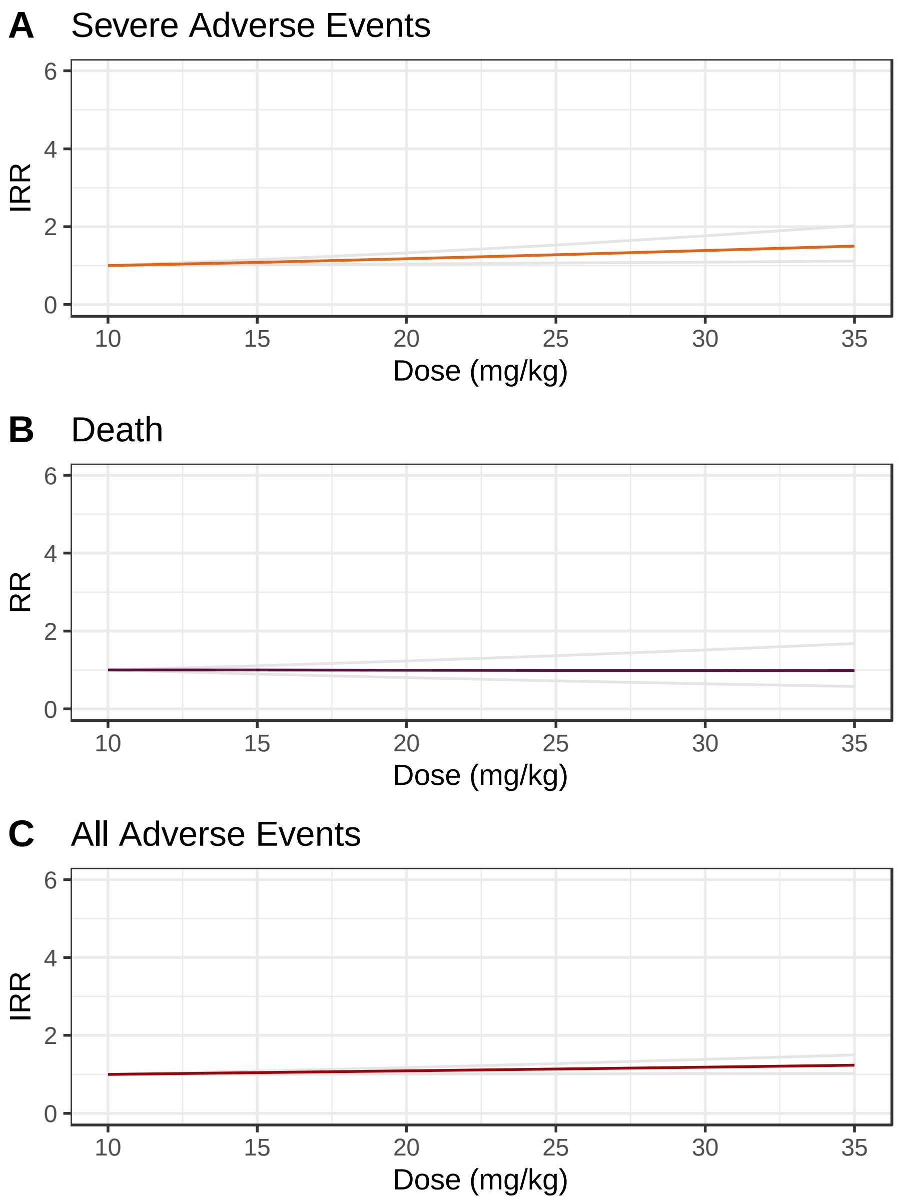

Supplementary Figure 2: Predicted outcome for dose response meta-analysis for three key outcomes, (A) total severe adverse events, (B) death, and (C) all adverse events. Ribbon represents 95% confidence interval. *Abbreviations*: IRR, incident rate ratio; RR, risk ratio.

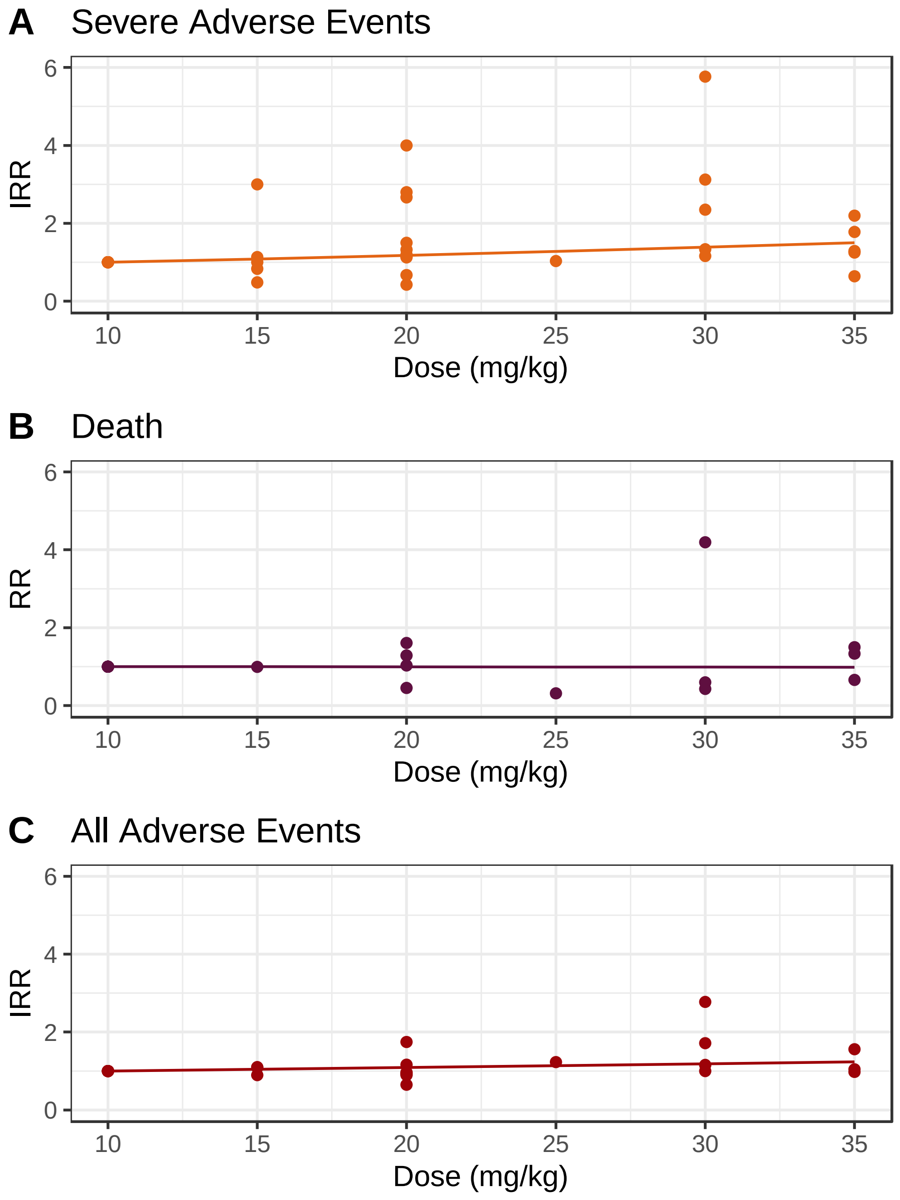

Supplementary Figure 3: Predicted outcome for dose response meta-analysis for three key outcomes, (A) total severe adverse events, (B) death, and (C) all adverse events, presented with raw data.
Abbreviations: IRR, incident rate ratio; RR, risk ratio.

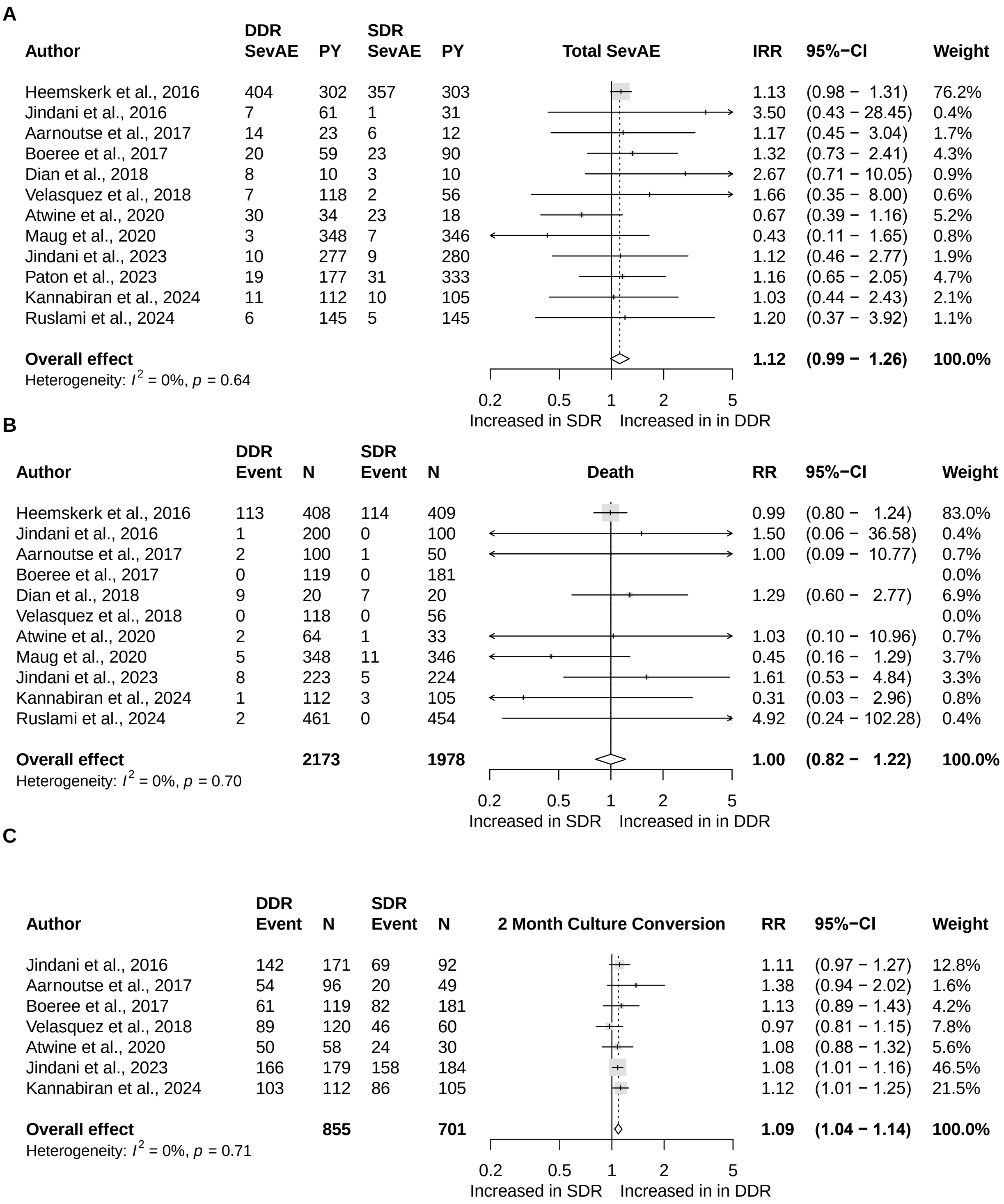

Supplementary Figure 4: Forest plots for subgroup analyses comparing double-dose rifampin to standard-dose rifampin on three secondary outcomes: (A) total severe adverse events, (B) death, (C) two-month culture conversion. *Abbreviations*: CI, confidence interval; DDR, double-dose rifampin, IRR, incidence rate ratio; PY, person-years;RR, risk ratio; SDR, standard-dose rifampin; SevAE, severe adverse events.

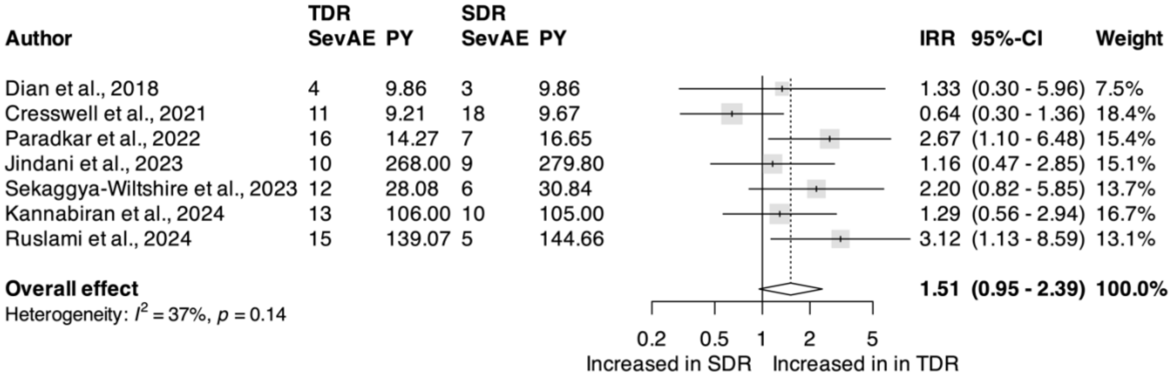

Supplementary Figure 5: Sensitivity analysis of total SevAE (primary outcome), restricting to studies in which only rifampin dose varied

Abbreviations: IRR, incident rate ratio; SDR, standard-dose rifampin; SevAE, severe adverse events; TDR, triple-dose rifampin.
